# Pathogenicity of duck plague and innate immune responses of the Cherry Valley ducks to duck plague virus

**DOI:** 10.1038/srep32183

**Published:** 2016-08-24

**Authors:** Ning Li, Tianqi Hong, Rong Li, Mengjiao Guo, Yao Wang, Jinzhou Zhang, Jiyuan Liu, Yumei Cai, Sidang Liu, Tongjie Chai, Liangmeng Wei

**Affiliations:** 1 College of Animal Science and Veterinary Medicine, Shandong Agricultural University, Sino-German Cooperative Research Centre for Zoonosis of Animal Origin of Shandong Province, 61 Daizong Road, Tai’an City 271000, Shandong Province, China; 2 Collaborative Innovation Centre for the Origin and Control of Emerging Infectious Diseases of Taishan Medical College, Tai’an City 271000, Shandong Province, China

## Abstract

Duck plague caused by duck plague virus (DPV) is an acute and contagious disease. To better understand the pathogenic mechanism of duck plague virus in ducklings, an infection experiment was performed. Our results showed that typical symptoms were observed in the infected ducklings. DPV could replicate quickly in many tissues, leading to pathological lesions, especially on the spleen. Real-time quantitative PCR demonstrated that expression of many innate immune-related genes was mostly up-regulated in the brain, and the antiviral innate immune response was established, but not sufficient to restrict viral replication. In contrast, although the expression of many major pattern recognition receptors (PRRs) increased in the spleen, the expression of most cytokines was declined. Our study indicates that DPV is a pantropic virus that can replicate rapidly in tissues, causing serious pathological lesions but the immune responses are different in the spleen and brain. To our knowledge, this is the first report to systematically explore the expression profiles of the immune genes in the DPV-infected ducks. Our data provide a foundation for further study of the pathogenicity of duck plague.

Duck plague (DP), also known as duck vial enteritis, is an acute and highly contagious disease in waterfowl such as duck and geese. The disease is currently common in many countries^1^,^2^,^3^ In China, it was first reported by Huang *et al*. in 1957^4^, and sporadic cases have been reported in many regions in recent years^2^,^5^. Considering that the disease can cause high morbidity and mortality in domestic waterfowl, it poses potentially great economic losses to the commercial waterfowl industry. Duck plague virus (DPV), the causative agent of the infectious disease, is an enveloped, double-stranded DNA virus, which belongs to the family *Herpesviridae*^6^.

The innate immunity is the first line of host defense against the pathogenic microbes and plays a key role in the induction of the adaptive immunity. Innate immunity is activated through pathogen recognition receptors (PRRs) which recognize the conserved microbial molecules, known as pathogen-associated molecular patterns (PAMPs), then the production of type I interferons (IFNs) and inflammatory cytokines are induced, and the innate immune response is established^7^,^8^. Among a variety of PRRs, one class of receptors called Toll-like receptors (TLRs), which are a group of conserved type I transmembrane proteins located on the plasma membrane or endosomal membrane, recognize PAMPs such as single- and double-stranded RNA (dsRNA), DNA, and lipopolysaccharide of Gram-negative bacteria^9^. Both DPV and herpes simplex virus (HSV) belong to the herpes virus, which have the CpG motif in their genomes. It has been demonstrated that HSV is first sensed by TLR2, which is expressed on the cell surface and recognizes viral glycoproteins^10^. TLR2 can induce the production of proinflammatory cytokines and chemokines in response to HSV^11^. In addition to TLR2, TLR9 has been reported to play an important role in the pathogenesis of HSV, as it can recognize the CpG motifs of HSV DNA and initiates the type I IFNs and cytokine secretion^12^,^13^. In recent years, Brownlie *et al*. demonstrated that TLR21 acts as a functional homologue to the mammalian TLR9 in response to microbial DNA^14^.

Recently, Friedemann *et al*. has reported that dsRNA accumulates in the HSV-infected cells^15^. Considering the existence of so many PRRs, it’s not surprising that pathogens are often recognized by multiple receptors. It is well known that TLR3 activate the antiviral immune responses through binding to dsRNA^16^,^17^. The results from Zhang *et al*. suggest that TLR3-mediated production of type I and II IFN may be crucial in the HSV infection^18^. Moreover, another class of PRRs, namely RIG-I like receptors (RLRs), including the retinoic acid-inducible gene I (RIG-I) and melanoma differentiation-associated gene 5 (MDA5), are expressed in the cytoplasm and primarily sense dsRNA or 5′-triphosphate single-stranded RNA^19^. Given that the replication of the viral genome leads to the production of intermediate dsRNAs, which are sensed by RIG-I and MDA5, RLRs interact with the IFN-β promoter stimulator 1 (IPS-1) adaptor protein upon recognition to induce the synthesis of the antiviral effectors and establish an antiviral state^20^,^21^,^22^.

Although many cases or outbreaks of DP have been reported^2^,^5^, and many studies of viral molecular biology have been published^23^,^24^, research about the pathogenicity of DPV in ducklings is still relatively sparse and the role of the host innate immune responses in these processes has not been extensively studied. Therefore, in the present study, clinical symptoms, pathological changes, and the viral distribution in the tissues of the ducks infected with DPV were analyzed, with a special focus on the innate immune response to this virus to systemically explore the pathogenicity of DPV in 21-day-old Cherry Valley ducks.

## Results

### Clinical symptoms and gross lesions

**Figure 1:**
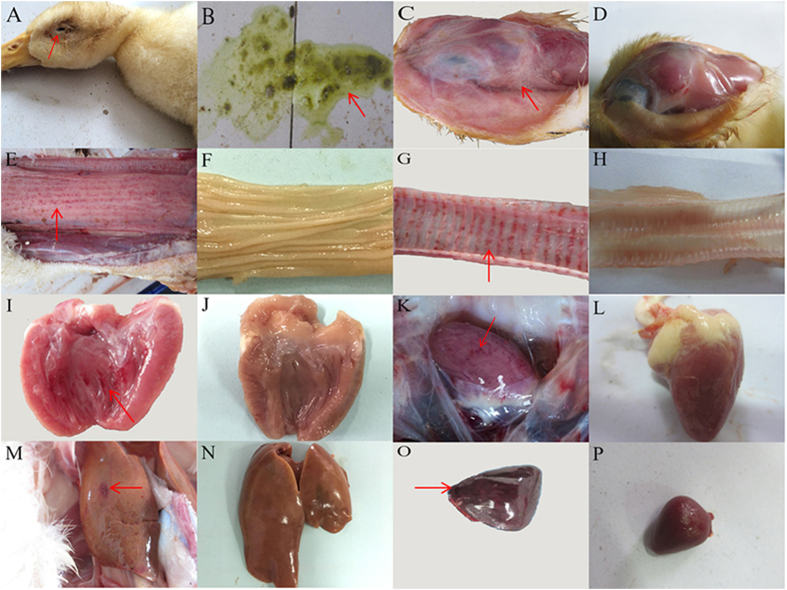
Clinical symptoms and gross lesions of the DPV-infected ducks. (**A**) Swollen head and neck; (**B**) Greenish diarrhea; (**C**) A light-yellow and transparent liquid of the head; (**E**) Diffuse hemorrhage of the esophageal mucosa; (**G**) Hemorrhage of the annulus trachealis; (**I**) Spotted hemorrhage of the endocardium; (**K**) Petechial hemorrhaging of the epicardium; (**M**) Liver is enlarged with blood spots; (**O**) Splenomegaly and hemorrhage. D, F, H, J, L, N, and P represent the head, esophagus mucosa, trachea, endocardium, epicardium, liver, and spleen of ducks from the control group, respectively.

All ducks from the infected group exhibited typical symptoms at 3–8 day post-inoculation (dpi), including depression, loss of appetite, dropping wing, retracted neck, swelling of the head and neck, swollen eyelids (arrow on Fig. 1A), and tearing, as well as greenish diarrhea (arrow on Fig. 1B). In addition, twelve severely infected ducks also showed dyspnea, ataxia, and paralysis. A yellowish and transparent liquid (arrow on Fig. 1C) was observed in the subcutaneous tissue of the swollen head and neck of the viral infected ducks. The diffuse hemorrhage of the esophageal mucosa and the annulus trachealis (arrow on Fig. 1E,G) was apparent, and hemorrhage of the endocardium and epicardium (arrow on Fig. 1I,K) was observed. Swelling and petechiae in the liver (arrow on Fig. 1M) and splenomegaly (arrow on Fig. 1O). Hemorrhage in bursa of Fabricius was serious (data was not shown). Out of the fifty ducks in the infected group, fourteen died of infection at 4–6 dpi. No clinical symptoms or gross lesions were observed in the ducks in the control group (Fig. 1D,F,H,J,L,N,P).

### Histopathological analysis

**Figure 2:**
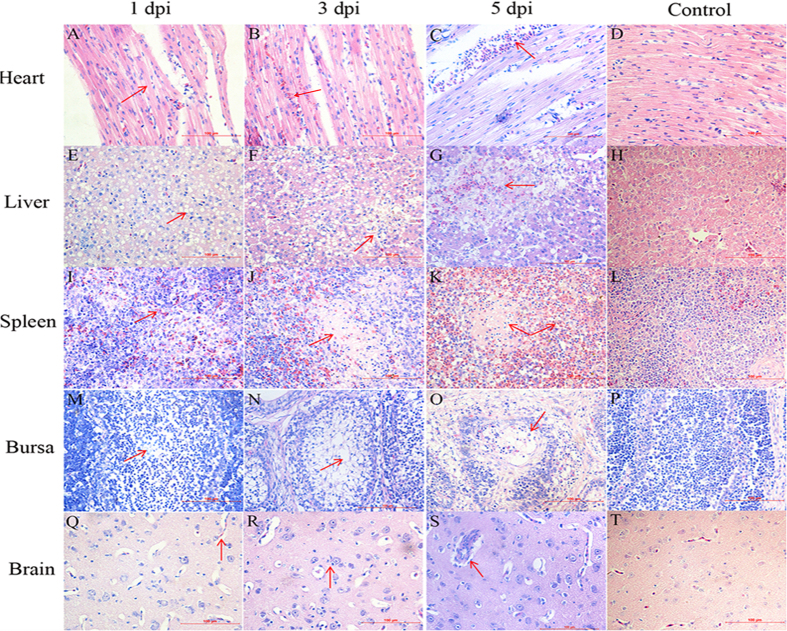
Pathological changes of the DPV-infected ducks at the different time points. (**A**) Mild granular degeneration of myocardial fibers at 1 dpi; (**B**) A small amount of erythrocyte infiltration at 3 dpi; (**C**) Myocardium haemorrhage at 5 dpi; (**E**) Fatty degeneration in the liver at 1 dpi; (**F**) Fatty degeneration and focal necrosis of hepatocyte at 3 dpi; (**G**) Hepatocyte necrosis with hemorrhage at 5 dpi; (**I**) Slight congestion in the white pulp of the spleen at 1 dpi; (**J**) Necrotic foci of lymphocyte at 3 dpi; (**K**) Lymphocytic necrosis with diffuse hemorrhage at 5 dpi; (**M**) Slight reduction of lymphocytes in the bursa of Fabricus at 1 dpi; (**N**) Lymphocytes dissolved and disappeared, the number decreased significantly at 3 dpi; (**O**) Serious necrosis of lymphocytes at 5 dpi; (**Q**) Brain edema at 1 dpi; (**R**) Neuronophagia of the brain at 3 dpi; (**S**) Perivascular inflammatory infiltrates at 5 dpi. D, H, L, P, and T represent the heart, liver, spleen, bursa of Fabricus, and brain of ducks from the control group, respectively. Magnification, ×400.

Pathological changes were detected in various tissues in the infected ducks and the lesions increased with time. Slight granular degeneration of myocardial fibers (arrow on Fig. 2A) was observed in the infected ducks at 1 dpi, and erythrocytes infiltration (arrow on Fig. 2B) at 3 dpi. At 5 dpi, significant hemorrhage (arrow on Fig. 2C) was observed. In the liver, liver cells displayed diffuse fatty degeneration (vacuolus of the same size in the liver, arrow on Fig. 2E) at 1 dpi, fatty degeneration and focal necrosis of hepatocytes (arrow on Fig. 2F) at 3 dpi, and hepatocyte necrosis with hemorrhage (arrow on Fig. 2G) at 5 dpi. A small amount of erythrocyte infiltration (arrow on Fig. 2I) in the white pulp of the spleen at 1 dpi, coagulated necrotic foci of lymphocyte (arrow on Fig. 2J) was found at 3 dpi, and obvious lymphocytic necrosis (nuclei became pyknotic and underwent rupture and karyolysis) with diffuse hemorrhage (arrow on Fig. 2K) at 5 dpi. Coincidently, serious histopathological changes were also observed in the bursa of Fabricus. A slight decrease in the number of lymphocytes (arrow on Fig. 2M) at 1 dpi, necrosis of lymphocytes and lymphocytes decreased significantly (arrow on Fig. 2N) at 3 dpi, at 5 dpi, microscopic lesions were serious and massive lymphocytic necrosis (many cells have dissolved and disappeared) (arrow on Fig. 2O). The lesions of the brain were slight compared to those of the spleen and bursa of Fabricus. The tissue edema (increasement of perivascular gap, arrow on Fig. 2Q) was found at 1 dpi, and neuronophagia (microglia proliferation with the phagocytosis of necrotic neurons, arrow on Fig. 2R) and perivascular inflammatory infiltrates (arrow on Fig. 2S) were detected at 3 and 5 dpi, respectively, suggesting a mild viral encephalitis during the infection. No microscopic lesions were observed in the control group (Fig. 2D,H,L,P,T). Overall, our results indicate that DPV could cause pathological lesions in a variety of tissues, specially the spleen and bursa of Fabricus with serious lesions, which indicated DPV might target the immune organs.

### Viral DNA load in different tissues of the DPV-infected ducks

**Figure 3:**
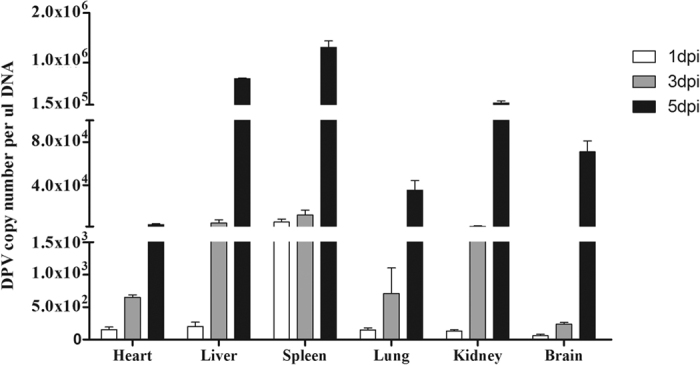
DPV replication in the tissues of the infected ducks. The data were expressed as means ± standard deviation, and five infected ducks were randomly selected for detecting the viral DNA load using the qRT-PCR method.

The viral DNA loads in a variety of tissues were measured by real-time quantitative PCR (qRT-PCR). As shown in Fig. 3, the viral DNA in the spleen increased to 5.73 × 10^3^ copies/μL at 1 dpi, while the viral DNA in other tissues were lower than 2.02 × 10^2^ copies/μL. At 3 dpi, the loads of DPV in various tissues were elevated and the viral content in the spleen still represented the highest among these tissues (1.22 × 10^4^ copies/μL), followed by the liver and the kidney. The viral load in the brain was the lowest (2.39 × 10^2^ copies/μL). At 5 dpi, the loads from all tissues were significantly increased, the viral titer in the spleen reached the peak (1.31 × 10^6^ copies/μL). Of note was that the viral load in the brain was 7.1 × 10^4^ copies/μL at 5 dpi, which indicates that DPV can quickly replicate in the central nervous system. Taken together, our results show that DPV could quickly invade and replicate in many tissues, including the central nervous system.

### Expression of innate immune-related genes in the brain and spleen of the DPV-infected ducks

**Figure 4:**
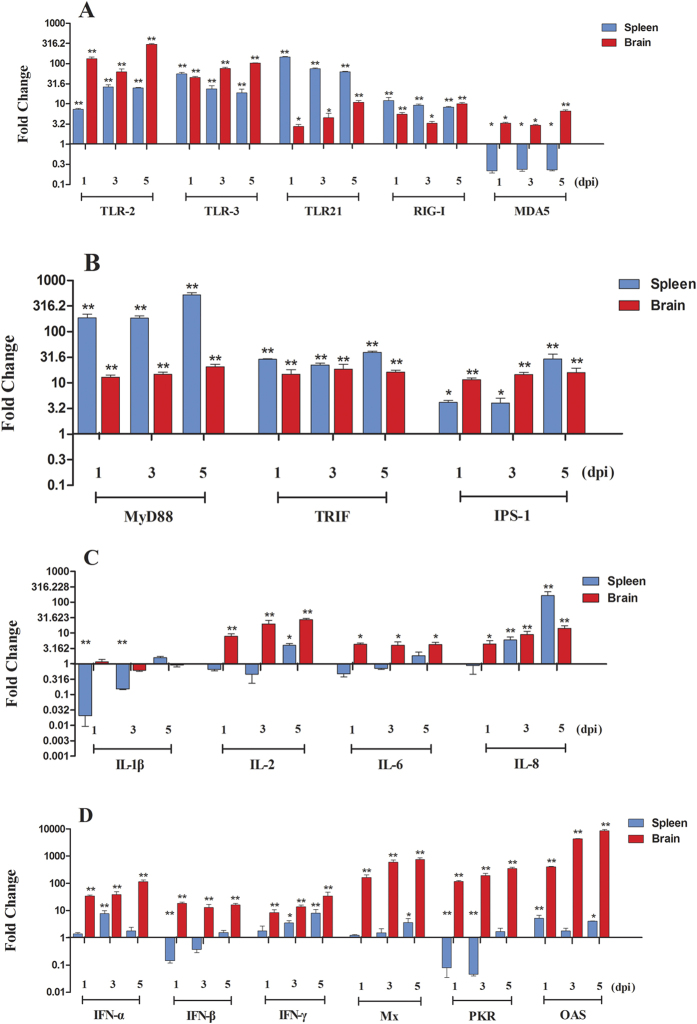
Expression profiles of PPRs in the spleen and brain of DPV-infected ducks. The samples of viral infected ducks were collected at 1, 3, and 5 dpi. Total RNA was extracted and cDNA was prepared for detecting the cytokines. The expressions of cytokines tested in this study were measured using 2^−ΔΔCt^ method by relative quantification. Data were expressed as mean fold change (n = 5). (**A**) The expression of major PRRs contained the TLR2, TLR3, TLR21, RIG-I, and MDA5; (**B**) The expression of relevant adaptor protein molecules contained the MyD88, TRIF, and IPS-1; (**C**) The expression of proinflammatory cytokines (IL-1β, IL-2, IL-6, and IL-8); (**D**) The expression of type I and II IFNs and ISGs (Mx, PKR, and OAS). Differences were analyzed with Student’s *t* test and were considered significant as follows: *P < 0.05; **P < 0.01.

Innate immunity is a conservative host defense system which is activated through PRRs. Expressions of five PRRs, namely TLR2, TLR3, TLR21, RIG-I, and MDA5, were examined in the brain and spleen. As shown in Fig. 4A, the results show that the expression of TLR2 was greatly up-regulated in both the brain and the spleen during the tested days, indicating that in these tissues TLR2 can recognize DPV through the CpG motif. Furthermore, the expression level of TLR2 was generally higher in the brain than in the spleen. For example, the expression was elevated by 132.60-fold in the brain at 1 dpi (*P* < 0.01; Fig. 4A), which is 18 times higher than that in the spleen. These results indicate the TLR2 pathway may play a more significant role in the brain than in the spleen. On the other hand, the TLR21 pathway may be involved mainly in the spleen, suggested by our data that the TLR21 expression increased more than 100 fold in the spleen at 1 dpi (*P* *<* 0.01), which is 50 times higher than that in the brain (2.76-fold, *P* < 0.05; Fig. 4A). The expression of TLR3 was elevated in both tissues (more than 40 fold; *P* < 0.01) at 1 dpi with no obvious tissue preference (Fig. 4A). The other two PRRs, namely RIG-I and MDA5, had a relatively low expression compared with TLR2 and TLR3, with a fold change between 3 to 12 fold in the brain or the spleen. In particular, the expression of MDA5 was suppressed by 5 fold in the spleen. These results indicate the engagement of the PRR pathways is tissue-dependent.

Signal transduction upon engagement of PRRs by PAMPs is mediated through a number of adaptor proteins. In this study, we examined a number of adaptor proteins for PRRs. As the adaptor protein of TLR2 and TLR21, MyD88 showed an elevated expression in both the brain and the spleen (Fig. 4B). Similarly, the Toll/IL-1 receptor domain-containing adaptor inducing (TRIF) and IFN-β promoter stimulator 1 (IPS-1), adaptor protein for TLR3 and RLRs, respectively, had an even higher expression in both tissues (Fig. 4B). These data suggest that the PRR pathways are activated post inoculation.

Upon PRRs pathway activation, a number of proinflammatory cytokines were induced, including IL-2, IL-6, IL-8 and IL-1β. However, there was a remarkable difference in terms of cytokine expression between the brain and spleen. As shown in Fig. 4C, the expression of IL-2 and IL-6 was up-regulated in the brain by 4-28 fold, while the expression change was relatively low in the spleen. The expression of IL-8 was elevated in both the brain and the spleen. At 5 dpi, the expression of IL-8 increased by 162.48-fold. However, the IL-1β expression in the brain was basically unchanged, whereas the expression of IL-1β in the spleen was suppressed by more than 40-fold at 1 dpi. Additionally, the activation of PRRs initiates the IFN-I system and many viruses can trigger the expression of IFN-I, which leads to the production of numerous IFN-stimulated genes (ISGs). We examined the expression of IFNs (IFN-α, IFN-β and IFN-γ) and three ISGs (Mx, PKR, and OAS). All of these genes showed elevated expression in the brain (Fig. 4D). The expression of IFNs in the brain increased by 8–117 fold, and the expression of ISGs showed similar tendencies, but to a greater extent (Fig. 4D). The expression of OAS increased 8515.33-fold at 5 dpi (Fig. 4D). However, in the spleen, the expression changes of these factors were not as obvious as in the brain, ranging from 1 to 10 fold (Fig. 4D). The expression of IFN-β and PKR, in particular, were suppressed by 10 fold at 1 dpi (Fig. 4D). Considering the anti-viral effects of the products of these ISGs, these data further explain the lower viral loads in the brain (Fig. 3).

Taken together, our results show that several PPRs were involved in the recognition of DPV, and they may cooperatively induce the expression of cytokines, IFNs and ISGs, might play a critical role in the process of fighting the DPV infection.

## Discussion

In the present study, we established an experimental model for DPV infection to investigate the pathogenicity of duck plague in Cherry Valley ducks. Our results show that the incubation period of duck plague was about 2 days, the whole course of the disease was about 7 days. DPV had broad tissue tropism in the infected ducks and replicated rapidly in a variety of tissues, especially the spleen and bursa of Fabricius, causing serious pathological lesions. Other report demonstrated that the levels of DPV in systemic organs had a close relation with the progression of the disease^25^. In the current study, we systematically studied the expression profiles of the innate immune-related genes in the brain and spleen. To our knowledge, this is the first report regarding the expression profiles of the innate immune-related genes in the DPV infected ducks.

Our results suggest that the TLR mediated immune responses are more important than that of RLRs based on the higher expression levels of TLRs (Fig. 4A). Among the TLRs, TLR21 may play a dominant role in the spleen, while TLR2 may be more important in the brain, indicating that the engagement of the PRRs pathways is tissue-dependent. Tissue-specific immune responses have been demonstrated in previous studies. For example, Hokeness-Antonelli *et al*. reported TLR9, a human homologue of TLR21, is the main pathway for the early immune response to murine cytomegalovirus in the spleen^26^. In the spleen, the cells are mainly lymphocytes, while in the brain, microglial cells are mainly involved in the innate immunity^27^. The difference of the cell types may lead to different immune response to DPV.

One important notion is that many proinflammatory cytokines and IFNs expression levels were not elevated in the spleen of the infected ducks, although several PRR pathways were activated (Fig. 4A,C). Many viruses have evolved mechanisms to evade the host immune response. For example, HSV can block the nuclear accumulation of interferon regulatory factor 3, which was required for the activation of type I IFN^28^. HSV can also facilitate the dephosphorylation of eukaryotic translation initiation factor-2α to influence the effects of dsRNA and IFNs on PKR, which in turn inhibits viral replication^28^. Here we speculate that DPV, which belongs to the same genus as HSV, also employs similar strategies to restrict innate immune response in the spleen at the early stages. The serious pathological lesions in the spleen might have contributed to the inhibition of cytokine expression. Similar results were obtained in a study by Volmer *et al*., in which the authors discovered that IL-1β, IL-8 and IFN-α were not induced, in the low pathogenic avian influenza H7N1-infected duck intestine^29^. The expression of the PRRs showed a significant correlation with the IFNs and ISGs in the brain, while this correlation was not existed in the spleen (Fig. 4D). This tissue-specific correlation is of interests and should be further investigated. Understanding the immune responses in different tissues after DPV infection may provide key insights of the immune pathways, which can be used for prophylactic or therapeutic purpose.

Another interesting finding in this study is the suppressed MDA5 expression by DPV infection in the spleen. By comparison, the expression of MDA5 increased ranging from 3 to 7 fold in the brain. MDA5 mRNA can be induced upon viral infection. In one study, Wei *et al*. showed the mRNA expression of MDA5 was up-regulated by 46 fold in the spleen of Muscovy ducks following-highly pathogenic avian influenza H5N1 infection^30^. Since PRRs were activated other than MDA5, it is not surprising that IPS-1, an adaptor protein of RLRs pathway, upregulated in the spleen and brain, the immune response through the MDA5 pathway may not play a decisive role. Cornelissen *et al*. revealed that the expression of MDA5 in the ducks infected with low pathogenic avian influenza H7N1 was not significantly increased in the lung^31^. Our data show that the expressions of TLRs were much higher, indicating these pathways through TLRs are the major responses upon viral infection. Nevertheless, understanding of the role of MDA5 during the DPV infection will provide valuable information towards elucidation of the mechanism of viral infection.

In this work, we presented a complex expression profile of PRRs, cytokines, interferons and ISGs. However, we did not address whether all the PRRs are responsible for the virus recognition. Further investigations, such as siRNA knockdown of the PRRs or poly (I:C) stimulation, should be carried out to better understand their exact roles in the DPV infection. Antiviral strategies against DPV infection may be developed based on the specific molecular pattern that triggers the PRR pathways. In summary, our results show that several PRRs are activated in the response to DPV, which supports the idea that a comprehensive innate antiviral response is initiated by many PRRs acting cooperatively to mediate host defense. However, the immune system responds differently in the brain than in the spleen. In the spleen, the IFN response is limited compared to those in the brain leading to a faster virus replication in the spleen. The opposite is true in the brain. As far as we know, this study is the first to systematically explore the expression profiles of the immune genes in the DPV-infected ducks, and the data will provide new insights into the pathogenesis of DPV.

## Materials and Methods

### Ethics Statement

The study was approved by the Committee on the Animal Ethics of Shandong Agricultural University. Experiments were carried out in accordance with the approved guidelines.

### Virus and animals

The DPV strain used in this study, namely the GM strain, was isolated from the clinical infected ducks in Weifang, Shandong province. The strain was donated by Liting Qin of New Hope Liuhe Co. Ltd, Qingdao, China. Virus stocks were propagated in duck embryo fibroblasts. The virus titer was determined to be a 10^6.5^ median tissue culture infective dose (TCID_50_)/mL by infection of duck embryo fibroblasts and the calculation of the titers followed the Reed and Muench method^32^.

One hundred one-day-old Cherry Valley ducks were purchased from a farm, and raised in isolators. Serum samples were detected by ELISA to verify that all ducks used in this study were free of DPV^33^.

### Experimental procedure

After three weeks, the ducks were randomly divided into two groups of fifty, and housed in separate isolators. The ducks from the first group were used for an infection experiment and received an intramuscular injection with 0.3 mL of virus stocks. The other group was set as the control group and the ducks were intramuscularly inoculated with 0.3 mL sterile phosphate-buffered saline. The related signs of DP such as swollen head, photophobia, tearing and mortality were observed for 21 days. On 1, 3, 5, 7, 9, 14 and 21 dpi, five ducks from each group were sacrificed, the organs (heart, liver, spleen, lung, kidney, and brain) were collected, and part of the tissues were fixed with 4% paraformaldehyde solution for histopathological examination while others were stored at −80 °C for RNA extraction. The rest of the ducks were euthanized at the end of this study through the intravenous administration of sodium pentobarbital (100 mg/kg body weight)^34^.

### Viral load in the tissues of the infected ducks

Viral DNA was extracted from the collected tissues (1 g) using the conventional method. In brief, 12.5 μL proteinase K and 50 μL 10% SDS were added into the samples (437.5 μL) for 30 minutes of water bath to dissolve samples and denature protein. Subsequently, phenol, chloroform, and other organic solvent were used to extract and purify the DNA, and each sample 15 μL of water was added at the end. The viral DNA load in the tissues was measured by qRT-PCR. The primers were designed as previously described^35^. qRT-PCR was performed on an Applied Biosystems 7500 Fast Real-Time PCR System (Applied Biosystems, CA, USA) with the SYBR Green PCR kit (Vazyme Biotech Co., Ltd., Nanjing, China). The PCR experiments were conducted in a total volume of 20 μL. The PCR reaction was initialized by a denaturing step at 95 °C for 5 min, followed by 40 cycles of amplification (95 °C for 10 s, and 60 °C for 34 s), and a dissociation curve analysis step. Each sample was analyzed in triplicate.

### The innate immune response of the infected ducks

qRT-PCR was employed to detect the mRNA expression of the innate immune related genes in spleens and brains from the experimental ducks, and their relative level was quantified using the previously reported primers^30^,^36^,^37^. The primers for the duck TLR2, TLR21, MyD88, TRIF, and IPS-1 genes were designed by the Primer3 software (http://bioinfo.ut.ee/primer3-0.4.0/), based on the published GenBank sequences (Table 1). The operation method and reaction conditions of qRT-PCR were described above.

### Statistical analysis

The relative expression mRNA of the target genes in the infected and control groups were calculated based on 2^−ΔΔCt^ method and quantified relative to glyceraldehyde-3-phosphate dehydrogenase (GAPDH), which was employed as the endogenous control to normalize the expression levels of the target genes. The fold changes were logarithmically transformed. All data were expressed as means ± standard deviation and were analyzed with Student’s t test processed by GraphPad Prism 5.0 (GraphPad Software Inc., San Diego, CA, USA). Statistical significance was set at *P* < 0.05 or *P* < 0.01.

## Additional Information

**How to cite this article**: Li, N. *et al*. Pathogenicity of duck plague and innate immune responses of the Cherry Valley ducks to duck plague virus. *Sci. Rep.* **6**, 32183; doi: 10.1038/srep32183 (2016).

## Figures and Tables

**Table 1:** Primers used in this study.

Primer name	Sequence(5′-3′)	Product size (bp)	GenBank no.
TLR2 F	AAGAAAATGGAGCTGCTGGA	231	NM001310348.1
TLR2 R	GAAAAACACAGCGCAGATCA		
TLR21 F	CCAAGAACGAGCTGAAGACC	157	NM001030558.1
TLR21 R	TAGGGGTAGACCACCTGCAC		
MyD88 F	TCCATCAGCGGAGAGCTTAT	168	NM001310832.1
MyD88 R	CTTCATGGCTTTGCACTTCA		
TRIF F	GCTTTCAGGATGCTTTGGAG	155	KJ466051.1
TRIF R	CTTGGGAACAAAAGGGATGA		
IPS-1 F	ACATCCTGAGGAACATGGAC	243	KJ466052.1
IPS-1 R	AGACCTCCTGCAGCTCTTCG		
